# Surgical outcomes of Tenzel rotational flap in upper and lower lid reconstruction without repair of posterior lamella: A modified approach

**DOI:** 10.22336/rjo.2021.70

**Published:** 2021

**Authors:** Salil Kumar Mandal, Anwesha Maitra, Purban Ganguly, Stuti Somani Agarwal

**Affiliations:** * Department of Orbit, Oculoplasty and Reconstructive Surgery, Regional Institute of Ophthalmology, Medical College and Hospital Calcutta, India

**Keywords:** lid, malignancy, reconstruction, rotational flap

## Abstract

**Aim:** To evaluate the efficacy and usefulness of Tenzel rotational flap in upper and lower lid reconstruction in malignant tumors, to determine the anatomical alignment, functional, and cosmetic outcome after lid reconstruction and recurrence of the tumor and to observe the canthal and fornix status.

**Methods:** Prospective, non-comparative,interventional clinical study of 30 patients over a period of 18 months. The study was conducted with strict inclusion and exclusion criteria. Evaluation parameters were LPS (Levator palpebrae superioris) action Pre and post operative, MRD-1 (Margin Reflex Distance-1) values pre and post operative, central vertical palpebral fissure height (PFH) pre and post operative,and the calculation of the created defect express in percentage preoperatively. None of the case posterior lamina was repaired. It formed or strengthened automatically.

**Results:** The diagnosis of Sebaceous cell carcinoma was 14/30 by FNAC and 17/30 by histopathology. The mean MRD-1 pre-operatively was -1.09 ± 1.54 mm to 4.02 ± 0.36 mm postoperatively in the upper lid. The mean LPS action pre-operatively was 4.35 ± 0.96 mm to 12.42 ± 1.24 mm postoperatively in the upper lid tumor. The mean pre-operative central palpebral aperture was 4.76 ± 0.88 mm and post-operative central palpebral aperture was 10.47 ± 0.88 mm in the upper lid tumor. The mean incision length was 8.16 ± 0.61 mm in upper lid and 9.27 ± 0.87 mm in lower lid Tenzel reconstruction respectively. In all the cases, posterior lamella grew normally. In addition, no strengthening procedure was required.

**Conclusion:** This reconstructive procedure was useful to repair 40%-60% of the upper lid and 40%-70% created a defect in the lower eyelid after the removal of the malignant tumor. Histopathological diagnosis of the specimen was more accurate than FNAC. No repair of posterior lamella was made. No extra surgical procedure was needed. Thus, the surgical procedure time was markedly reduced. After the surgery, all the patients presented a good uplift of the upper lid, good closure of the eyelids, and were visually and functionally well rehabilitated. The aesthetic appearance was excellent.

## Introduction

Lid reconstruction surgeries pose a great challenge for ocular plastic surgeons because they demand the best possible function, as well as cosmetic, outcome. Early reconstructive surgeries, aimed at simply closing the defect primarily, were the ones mentioned by Celsus in 25 B.C.- 50 A.D. [**1**]. In India, Sushruta first described the eyelid and nose reconstruction in a case of mutilated individuals as a form of punishment, which has been described explicitly in his work [**2**-**4**]. Full thickness defects of the eyelids that cannot be closed directly are repaired with flaps of local tissue [**5**,**6**]. Flaps are basically tissue lifted from a donor site and moved to the recipient site, with an intact blood supply [**7**-**9**]. This is performed to fill a defect such as a wound resulting from injury or surgery when the remaining tissue is unable to support a graft, or to build a more complex anatomical structure [**10**,**11**].

## Methods

To determine the efficacy of the Tenzel Rotational flap reconstruction of the upper and lower eyelid defects after the removal of large tumors without support of posterior lamina, to judge the restoration of the anatomical, functional, and aesthetic outcome after lid reconstruction and to evaluate the recurrence of the malignant tumor, canthal, and fornix status during long term follows up. This was prospective, non-comparative interventional study of 30 patients, over a period of three years, from January 2016 to December 2019. Ethical committee of Medical College, Kolkata, approved this study and an informed written consent was obtained from each patient, which was in line with the tenets of the declaration of Helsinki. In this study, the inclusion criteria were: all malignant eyelid tumors, created defect >40% to <60% of both the eyelid, FNAC and biopsy proved malignant tumors. The exclusion criteria were the following: ipsilateral eyelid tumors with other eyelid involvement, eyelid tumors with intra orbital extension, with local lymph node involvement,distant metastasis and cases with corneal anesthesia or gross corneal involvement or infiltration. Then, the mass was examined for its size, mobility, corrugated or pearly appearance, to roll out margins, pigmentation, ulceration, irregular margins, and any other associated characteristics were noted.

The parameters recorded were pre- and post-operative LPS action, Margin Reflex Distance-1 (MRD-1), eyelid closure, central vertical palpebral fissure height, upper eyelid created defect. The created defect was calculated and expressed in percentage. Mechanical ptosis was observed in this study due to the gravitational force. Such a ptosis is corrected after a definitive surgery.

## Surgical procedure

**Fig. 1:**
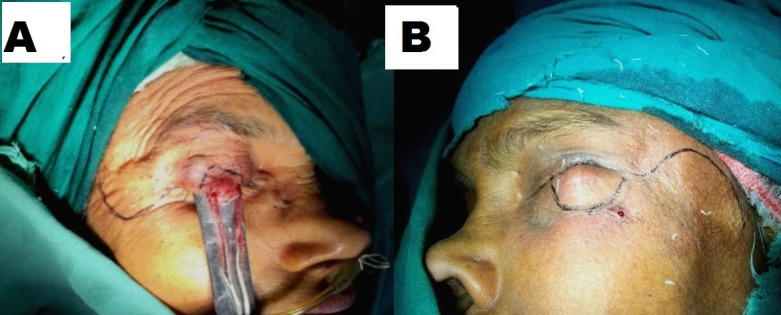
**A.** Incision pattern of upper lid Tenzel reconstruction flap. **B.** Incision pattern of lower lid Tenzel reconstruction flap.

**Fig. 2:**
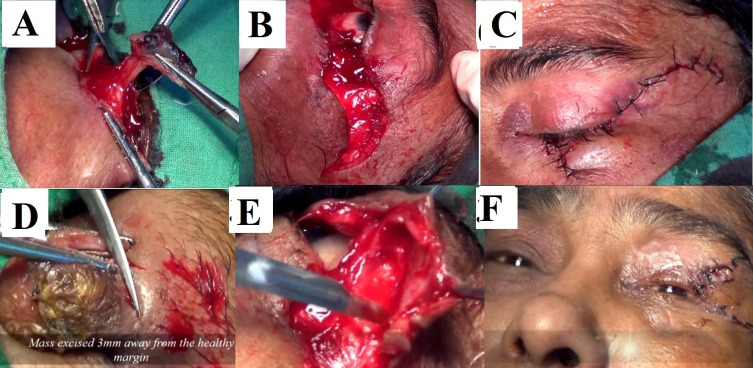
**A** Lower lid tumor excised with pentagon incision. **B.** Tinsel semicircular flap made a convexity upwards incision over the zygomatic temporal region. **C.** Closure of the wound in lower lid Tenzel reconstruction. **D.** Upper lid tumor excised with pentagon incision. **E.** Tinsel semicircular flap made a convexity downwards incision over the zygomatic temporal region. **F.** Closure of the wound in upper lid Tenzel rotational flap

This procedure was performed under general anesthesia. Incision line was marked first. It went 3-4 mm beyond healthy skin margin all around the mass, and marked in a pentagon shape encircling the tumor mass (**Fig. 1 A, B**). In each case per operative, frozen section biopsy was mandatory to exclude tumor free margin and to prevent postoperative recurrence (**Fig. 2 A, D**). A full thickness skin incision was made to create the flap, which began at the lateral canthus in a semi-circular fashion, arching the convexity upwards for the lower lid and convexity downwards for the upper eyelid. On Tenzel’s flap for lower eyelid, the incision began at the lateral canthus and curved upwards temporally, in a semicircular fashion, carried downwards, up to the tragus height of the incision, which never crosses the line joining the eyebrow to the upper border of the tragus. The lateral canthal ligament was excised at the lateral orbital rim to create a skin and muscle flap. That flap was prepared at the temporal arterial base from the skin, muscle, and the underlying bone, so that it became mobile. Lateral canthotomy and cantholysis was performed simultaneously, after the flap dissection was done, to mobilize and allow medial rotation of the flap (**Fig. 2 B, E**). The primary defect was closed in 2 layers, muscle and soft tissue and skin. The skin muscle flap was fixed at the lateral orbital margin over the pristine of the zygomatic bone, with 4-0 prolene sutures, to prevent the reversion of the flap to its original position. The new lateral canthus was recreated by suturing the deep fibers of the orbicularis oculi to the periosteum at the lateral orbital margin. The first suture was made at the tarsal plate, which was reinforced by skin sutures at the cut margin of the eyelid, as the suture line tension was the highest. Our objective was to achieve maximal anatomical continuity at the eyelid margins. Then, the wound was closed with interrupted sutures, using 5-0 polyglactin a delayed absorbable sutures. The overlying skin defect was closed similarly using 5-0 black silk (**Fig. 2 C, F**). It should be noted that no effort was made to suture or approximate the posterior lamella. Posterior lamella was neither created nor reconstructed, but it was observed that it was formed automatically along with smooth conjunctiva covering. In Tenzel’s Myo-cutaneous flap for the upper eyelid reconstruction, the incision began at the lateral canthus curves inferiorly, in a semicircular fashion, with a convexity downwards. The rest remained the same as above. In the postoperative period, patients were advised to blink frequently, but not overstrain. Graded stretching of the lateral canthus was advised, to achieve near normal eyelid apertures. Follow up was done at 1 month, 6 months and 12 months. Statistical analysis was performed with the help of Epi Info (TM) 3.5.3. EPI INFO is a trademark of the Centers for Disease Control and Prevention (CDC).

## Results

Pearson’s Goodness of fit test showed that the proportion of patients in the age group 56-65 years was 60%, which was significantly higher than the other age groups (p=0.00012). 20% of the patients were in the age group 46-55 (**Table 1**). The male patients were higher (60%) than the females (40%) (**Table 1**). The mean age of males was 55.92 ± 7.97 years. The mean age for females was 52.67 ± 11.24 years. The upper eyelid was involved in 70% of the cases, whereas the lower eyelid was involved in 30% of the cases. Fine needle aspiration study shows Sebaceous cell CA 46.67%, Basal cell CA 26.67%, Vascular lesion 6.67%, Squamous cell CA 6.67%, Chalazion 6.67% (**Table 2**). In this study, H.P. diagnosis showed Sebaceous cell CA (17) 56.67%, Basal cell CA (8) 26.67%, Squamous cell CA (2) 6.67%, Vascular tumor (2) 6.67%. Nodular hidradenoma (1) 3.33%, Chalazion nil respectively (**Table 3**). Therefore, the study showed that the diagnosis of Sebaceous cell CA was 14/ 30 by FNAC and 17/ 30 by histopathology. Similarly, sebaceous cell CA diagnosed by FNAC was chalazion, but on histopathology, it was a Sebaceous cell CA. It was proved that the histopathological diagnosis is much more reliable than FNAC (**Table 4**). The mean preoperative MRD-1 in this study was -1.04 ± 1.56 mm, with the median being -2 mm in the upper lid (n=21), and the mean preoperative MRD-1 value being 3.61 ± 0.33, with a median of 3.5 mm in the lower lid (n=9). The post-operative mean MRD-1 was 4.02 ± 0.29 mm and the median was 4 mm in the upper lid. The mean MRD-1 was 4.03 ± 0.35 mm, with a median of 4 (n=9) in the lower lid, which was statistically significant, p=0.0001 with t value -14.90. The mean preoperative LPS action was 4.35 ± 0.96 mm, median 4 mm in the upper lid tumors (n=21) and of a mean 11.44 ± 1.2 mm, with a median of 12 in the lower lid tumors (n=9). The mean post-operative LPS action was 12.42 ± 1.24 mm, median 12 mm in the upper lid tumors (n=21), which was statistically significant, *P*= 0.0001 with *t* value -23.12. In the lower lid, post operative means LPS action was 12.50 ± 0.77 mm, with a median of 12 at the end of 12 months. The mean pre-operative central palpebral fissure height was 4.76 ± 0.88 mm, with the median of 5 mm. The mean post-operative central vertical palpebral fissure height was 10.47 ± 0.88 mm, with the median of 10.55 mm in the upper lid tumor (n-21), which was statically significant, *P*=0.0001 with *t* value -22.12. In a lower lid tumor, post operative central vertical aperture was 10.33 ± 0.50, with a median of 10 (n=9). In this study, the mean incision lengths were 8.16 ± 0.61 mm, with a median of 8 mm in the upper lid Tenzel reconstruction and a mean incision length of 9.27 ± 0.87 mm, with a median of 9 mm respectively in the lower lid Tenzel reconstruction. Some of the complications in this study were conjunctival overgrowth 3 case, lid notching - 2 cases, need for secondary sutures - 1 case.

**Tabel 1:** MRD-1 pre and postoperative, LPS action pre and post operative, central vertical palpebral fissure height, pre and post operative, created defect expressed in percentage, length of incision expressed in mm

Sl. No.	MRD-1 Pre op. in mm	MRD-1 Post op. in mm after 6 months	LPS Action Pre op. in mm	LPS Action Post op. in mm after 6 months	Central Vertical fissure height in mm Pre op.	Central Vertical fissure height in mm after 6 months post op.	Created Defect in %	Length of incision in cm Convexity downwards in mm
				*Upper lid Tumors in Tenzel rotational flap for upper lid reconstruction*				
1.	+1	4	6	14	4	12	16÷30×100 =53.55%	9
2.	-2	4	4	15	2	13	17÷30×100 =56.66%	8
3.	-3	3.5	5	12	5	10	17÷30×100 =56.70%	8.5
4.	-3	4.5	5	12	5	10	17÷30×100 =56.70%	8.5
5.	+1	3.5	6	14	6	10.5	17÷30×100 =56.70%	8.5
6.	-2	4	3	12	4	11	12÷30×100 =40%	9
7.	-2	4	4	13	4	10	12÷30×100 =40%	9.5
8.	-1	3.5	5	12	5	10.5	19÷30×100 =63.30%	8.5
9.	-2	4	3	13	4	11	18÷30×100 =60%	8.5
10.	-4	4	5	15	3	12	15÷30×100 =50%	7
11.	-2	4	4	12	5	12	14÷30×100 =46.70%	7.5
12.	0	4	5	11	6	11	19÷30×100 =63.30%	8.5
13.	-2	4	3	10	4	10	15÷30×100 =50%	8
14.	+1	4	6	11	5	9	17÷30×100 =56.70%	8.5
15.	0	4	4	14	6	10	14÷30×100 =46.70%	7.5
16.	+1	4.5	4	13	6	10.5	16÷30×100 =53.30%	7.5
17.	+1	4	5	12	6	10	12÷30×100 =40%	8
18.	0	4.5	4	13	5	10.5	14÷30×100 =46.70%	7.5
19.	-2	4	4	12	4	11	16÷30×100 =53.30%	7.5
20.	-2	4.5	3	11	4	9	18÷30×100 =60%	8.5
21.	0	4	4	14	6	10	14÷30×100 =46.70%	7.5
				*Lower lid tumors in Tenzel rotational flap for lower lid reconstruction*				
22.	3.5	4	14	14	12	12	20÷30×100 =66.66%	10.5
23.	3.5	4	12	12	8	10	16÷30×100 =53.30%	10
24.	3.5	4	13	13	7	11	16÷30×100 =53.30%	9
25.	4	4.5	12	12	8	10	17÷30×100 =56.70%	8.5
26.	3.5	4	13	13	8	10	16÷30×100 =53.30%	9
27.	4	4	12	12	8	11	18÷30×100 =60%	10
28	3.5	4	10	12	9	10	13÷30×100 =43.30%	8
29	4	4.5	10	12	8	11	14÷30×100 =46.70%	8.5
30	3.5	3.3	10	10	8	10	19÷30×100 =63.3%	10
*MRD = Margin Reflex Distance, LPS = Levator palpebrae superioris, Pre-op = Pre-operative, Post op = Post operative, MM = Millimeter, PFH = Palpebral Fissure height*								

**Tabel 2:** Distribution of the diagnosis by FNAC of the specimen

Sebaceous cell CA	14	46.67%
Basal cell CA	8	26.67%
Squamous cell CA	2	6.67%
Infected epidermal cyst	2	6.67%
Vascular lesion	2	6.67%
Chalazion	2	6.67%

**Tabel 3:** Distribution of histopathological diagnosis of the specimen

HPE DIAGNOSIS	NUMBER (n=30)	PERCENTAGE%
Sebaceous cell CA	17	56.67%
Basal cell CA	8	26.67%
Squamous cell CA	2	6.67%
Vascular tumor	2	6.67%
Nodular hidradenoma	1	3.33%
Chalazion	0	0%

**Tabel 4:** Comparison of the diagnosis by FNAC and histopathology

DIAGNOSIS	FNAC	HISTOPATHOLOGY
Sebaceous cell CA	14	17
Basal cell CA	8	8
Squamous cell CA	2	2
Vascular tumor	2	2
Infected epidermal cyst	2	0
Chalazion	2	0
Nodular hidradenoma	0	1

## Discussion

Reconstruction of eyelid defects is difficult due to the complex anatomy and pose a challenge for the oculoplastic surgeon to reconstruct, aiming for both cosmetic and functional outcome. Improper reconstruction can cause serious problems like conjunctivitis, keratitis, entropion, ectropion, and poor aesthetic appearance. The complication rate of upper eyelid defect is higher than the lower eyelid.

Numerous procedures have been defined for the reconstruction of anterior lamella and posterior lamella. In this study, we discussed anterior lamella reconstruction with a rotational flap, without posterior lamella reconstruction. The advantages of a flap are that they carry their own blood supply and thus, they heal faster. Skin flaps contract lesser than the skin grafts after transfer. They are providers for the same color, texture, and surface characteristic. Adnexal structures have a better chance of survival and thus, they contribute to further matching to the surrounding skin. Flaps avoid additional surgery at a remote site [**12**,**13**]. They can be easily mobilized due to the slight elastic nature of the skin. Flaps can be of varied depths, and skin grafts however, need to have a minimum thickness for survival. Lastly, the flaps derive their nourishment from the underlying connective tissue and muscle. There is no dependence on any specific blood vessel for nourishment. Myocutaneous flaps have an additional muscular, vascular component - they can retain the muscular function [**14**,**15**]. Eyelid flaps, especially in the lower eyelid, are of two types - the advancement flaps and the switch flaps. The advancement flaps move about a pedicle. They are the following: tarsoconjunctival flap, cheek advancement flap, tripper bipedicle flap, Tenzel flap [**16**-**18**]. Tenzel’s semicircular rotational flap is a musculocutaneous flap, mobilized from the cheek, rotated around the lateral canthus, with an arch superiorly. It is used in lower eyelid defects >40% to <60% defects [**19**,**20**].

The experience of multiple institutions, including our own’s, highlights the excellent cosmetic result. In 1978, Tenzel RR published an article on eyelid reconstruction by the semicircular flap technique. The procedure combined with the use of selective lysis of the limbs of the lateral canthal tendon with a semicircular skin-muscle flap confined to the region of the lateral canthus, within the boundary of the lateral eyebrow and the arc it defined. The procedure has been used in the reconstruction of 36 lower eyelids and 5 upper eyelids in 41 patients, with a follow up period of 6 months to 6 years, in which the early and long-term cosmetic and functional results have been gratifying. Similar functional and cosmetic outcome was observed in our study, for a follow up period of 6 to 12 months.

In a retrospective study conducted by Anuradha Pradhan et al., in 2015, 30 patients underwent Tenzel’s eyelid reconstruction, between January 2015 and December 2015. Out of 30, 13 flaps were performed for upper eyelid defects and 17 flaps for lower eyelid defects. The cosmetic outcome was found to be acceptable along with a good lid stability.

In a retrospective study by Yordanov YP et al., in 2017, 13 patients with full thickness lower eyelid defects underwent Tenzel‘s reconstruction as a single stage procedure. It was found that females accounted for 9 cases and males for 4 cases. The mean age was 66.5 years. The most common malignancy was Basal cell carcinoma (n=11). These patients were followed up for 9 months. No additional intervention or second surgery was required in these patients [**21**-**23**].

In another retrospective study, performed in 2011, in the Department of Ophthalmology of Yonsei University in Korea and in Zhejiang University in China, on cases of confirmed malignant eyelid tumors, a total of 75 patients were analyzed, of whom 41 were males and 34 were females. The age of the patients ranged from 13 to 92. The upper eyelid tumors were found in 35 patients, the lower eyelid tumors in 32 patients, and less frequently in the canthus (both medial canthus and lateral canthus) in 8 patients. The mean age at diagnosis was 62.4 years (range 13-92). 

The study undergone by Dr. Abdur Rahman, in Yurtaslan Oncology Training and Research Hospital, Yenimahalle, Ankara, Turkey, comprised a total of four patients, with histopathologically confirmed upper eyelid malignant tumors, which were examined and treated. The patients were 60-73 years old and their average age was 66 ± 11.10 years. Our study concluded that the mean age of the patients was 54.1 ± 9.49 years and the median was 57.5 years. In our study, the male: female ratio was 1.5:1. The study showed that the proportion of male (n =18; 60%) patients was higher than the females [**24**,**25**].

In our study, the ratio of upper: lower lid involvement was 2.33:1. The upper lid was involved in 70% (n= 21) and the lower lid in 30% (n= 9) of the cases. This showed that there was a statistically significant chance of the involvement in malignancy of the upper lid. In our study, sebaceous cell carcinoma was diagnosed in 46.67% by FNAC and 56.67% with HPE, which was found to be statistically significantly higher than the other malignancies (p <0.05). On FNAC, the most common malignancy was found to be sebaceous cell carcinoma (n= 14), accounting for 46.67% of the cases, followed by basal cell carcinoma 26.67% (n= 8), squamous cell carcinoma, infected epidermal cyst, vascular hemangioma and Chalazion accounting for 6.67% each (n=2). On HPE, the most common malignancy was sebaceous cell carcinoma occurring in 56.67% (n=17), basal cell carcinoma 26.67% (n=8),squamous cell carcinoma 6.67% (n=2), vascular hemangioma 6.67% (n=2) and nodular hidradenoma 3.33% (n=1). Further, sebaceous cell carcinoma was the most common malignancy in the upper eyelid (n=13), 61.9% of upper eyelid cases and basal cell carcinoma was the most common malignancy of the lower eyelid (n=4), 44.44% of lower eyelid cases The study by Yordanov YP, undergone in 2017, on the reconstruction of skin malignancies after ablation, concluded that Tenzel’s reconstruction as a single stage procedure, for full thickness defects up to 60%, was a reliable option [**25**,**26**]. Our study concluded that created eyelid defects in the range of 40-66.70% can be successfully reconstructed with Tenzel’s rotational flap procedure with anterior lamellar reconstruction only in both upper and lower lid. Moreover, it is a single stage procedure. No such support of posterior lamella is required for both upper and lower lid reconstruction. The mean eyelid defect created was 51.44 ± 8.10 % (**Table 1**). Thus, this study concluded that single stage local flaps used in Tenzel’s reconstructive surgery were a gold standard option for lower eyelids, as they were highly reliable, with satisfactory results. Traditionally, reconstruction of conjunctival mucosa was done by another mucosa, hard palate, nasal mucosa, or it would cause corneal abnormalities [**27**-**29**]. All these aspects were taken into consideration in our study. The salient features were adequate mobilization of the skin, muscle flap to avoid any tension on the suture line of the newly reconstructed flap. Care was taken to avoid any excessive increase in the width to length ratio, as this could have led to a compromised vascularity. Further, no posterior lamella reconstruction was done in any case in our study, of even up to 66.70% created the defect. The tumor was resected with the incision line gone beyond 4-3 mm healthy tissue all around. A normal conjunctival growth was noted, even without any other mucosal supplementation. The fornix status was healthy and maintained throughout the follow-up period. No such contracture, symblepharon of forniceal conjunctiva was noted, as seen in the study by Smith RJ, in 2008. None of the patients developed any corneal abnormality [**30**-**32**]. Lid laxity was counteracted by fixing the flap to the lateral orbital periosteum. The new lateral canthus was recreated by suturing the deep fibers of the orbicularis oculi to the periosteum overlying the zygomatic bone.This was done to prevent the derotation of the flap in default flap setting. No retraction of the reconstructed eyelid was noted. In our study, the mean MRD 1 preoperatively was -1.09 ± 1.54 mm and the mean MRD 1 post-operatively was 4.02 ± 0.29 mm (**Table 1**). Though, it was a single stage surgery, thus, visual, and functional rehabilitation occurred much faster. In this study, it was observed that even after the removal of 66.5% of the upper lid along with LPS, no ptosis developed. In our study, another important parameter was the central palpebral fissure height. The mean pre-operative central palpebral fissure height was 4.76 ± 0.88 mm. The mean post-operative central vertical palpebral fissure height was 10.47 ± 0.88 mm, within the upper lid tumor (n-21). The mean pre-operative central palpebral fissure height was 8.26 ± 2.116 mm and post operative, it was 9.53 ± 0.97 mm. The post-operative improvement of central palpebral aperture was statistically significant, with a p value of 0.0001, with t -22.11, which is < 0.05. in the upper lid. Temporal scar mark was the most common complication seen in a retrospective study by Callahan MA and Callahan A, in 1980, followed by lid instability, sagging of lateral canthus and lid dehiscence. Others were: entropion, ectropion, conjunctival scarring, and corneal injury. In our study, the complications were conjunctival overgrowth in 3 case, which subsided with graded stretching at the lateral canthus. Notching of the upper eyelid was noted in 1 case, in a middle-aged male, in whom the created defect was 60%. This was due to lesser skin laxity in young as compared to elderly [**33**-**35**]. Wound dehiscence at the site of primary closure occurred in the 1 case and it required secondary suturing. In our study, there was no recurrence, although the influencing factors like larger lesions, histopathologic features, such as poor differentiation, multicentric origin, pagetoid spread, were similar to those of the previous study.

## Conclusion

The anterior lamella is important as it provided the skin coverage and blood supply to the eyelids. The posterior lamella provides a semi-rigid support and a non-abrasive mucosal surface. Posterior lamella reconstruction is not essential in Tenzel’s semicircular rotational flap, which takes advantage of the pre-existing skin laxity, especially in the elderly, along with cantholysis, allowing an adequate mobilization of the flap. In addition, there is no extra surgical step in Tenzel flap reconstruction. Thus, the surgical operative time is markedly reduced. This reconstructive procedure was useful in closing moderate sized upper (40%-60%) and lower (40%-70%) eyelid defects. Flaps are basically tissue lifted from an adjacent site and moved to the reconstructed site, with an intact blood supply. They provide a better reconstruction than skin grafts as they heal faster, contract lesser, provide for same color and surface characteristics. Adnexal structures have a better chance of survival. Flaps avoid additional surgery at a remote site. The reconstructed eyelid is freely mobile enough to protect the eyeball. Histopathological diagnosis of the specimen is superior to FNAC. After the surgery, all the patients had good uplift of upper lid, satisfactory closure of the eyelids, and were visually and functionally well rehabilitated. Aesthetic appearance was excellent.

**Conflict of Interest statement**

The authors state no conflict of interest.

**Informed Consent and Human and Animal Rights statement**

Informed consent has been obtained from all individuals included in this study.

**Authorization for the use of human subjects**

Ethical approval: The research related to human use complies with all the relevant national regulations, institutional policies, is in accordance with the tenets of the Helsinki Declaration, and has been approved by the review board of Department of Orbit, Oculoplasty and Reconstructive Surgery, Regional Institute of Ophthalmology, Medical College and Hospital, Calcutta, India.

**Acknowledgements**

Many thanks to all the Doctors of the Medical College, Calcutta, Department of Ophthalmology, Operation Theatre, and nursing staff, who contributed significantly to this study. Finally, yet importantly, many thanks to my teacher, Professor James Christian Fleming, MD, PhD, from Hamilton Eye Institution, Memphis, United States of America, who encouraged me to perform this Tenzel flap reconstruction for lid reconstructive procedure.

**Sources of Funding**

All the authors involved in this study had no conflict of interest and did not receive funding from any source. No special grant was allotted for this study.

**Disclosures**

None.
